# Possible Sexual Transmission of Ebola Virus — Liberia, 2015

**Published:** 2015-05-08

**Authors:** Athalia Christie, Gloria J. Davies-Wayne, Thierry Cordier-Lasalle, David J. Blackley, A. Scott Laney, Desmond E. Williams, Shivam A. Shinde, Moses Badio, Terrence Lo, Suzanne E. Mate, Jason T. Ladner, Michael R. Wiley, Jeffrey R. Kugelman, Gustavo Palacios, Michael R. Holbrook, Krisztina B. Janosko, Emmie de Wit, Neeltje van Doremalen, Vincent J. Munster, James Pettitt, Randal J. Schoepp, Leen Verhenne, Iro Evlampidou, Karsor K Kollie, Sonpon B. Sieh, Alex Gasasira, Fatorma Bolay, Francis N. Kateh, Tolbert G. Nyenswah, Kevin M. De Cock

**Affiliations:** 1 CDC; 2 World Health Organization; 3 Ministry of Health and Social Welfare, Liberia; 4 US Army Medical Research Institute of Infectious Diseases; 5 National Institutes of Health; 6 Médecins Sans Frontières; 7 Liberian Institute for Biomedical Research

On March 20, 2015, 30 days after the most recent confirmed Ebola Virus Disease (Ebola) patient in Liberia was isolated, Ebola was laboratory confirmed in a woman in Monrovia. The investigation identified only one epidemiologic link to Ebola: unprotected vaginal intercourse with a survivor. Published reports from previous outbreaks have demonstrated Ebola survivors can continue to harbor virus in immunologically privileged sites for a period of time after convalescence. Ebola virus has been isolated from semen as long as 82 days after symptom onset and viral RNA has been detected in semen up to 101 days after symptom onset (1). One instance of possible sexual transmission of Ebola has been reported, although the accompanying evidence was inconclusive (2). In addition, possible sexual transmission of Marburg virus, a filovirus related to Ebola, was documented in 1968 (3). This report describes the investigation by the Government of Liberia and international response partners of the source of Liberia’s latest Ebola case and discusses the public health implications of possible sexual transmission of Ebola virus. Based on information gathered in this investigation, CDC now recommends that contact with semen from male Ebola survivors be avoided until more information regarding the duration and infectiousness of viral shedding in body fluids is known. If male survivors have sex (oral, vaginal, or anal), a condom should be used correctly and consistently every time (4).

On March 14, 2015, a woman from Monrovia aged 44 years (patient A) developed headache, weakness, joint pain and nausea. She went to a hospital on March 19, and was triaged as a suspected Ebola patient to a nearby transit center (a facility for rapid isolation, diagnosis, and referral of Ebola patients). On March 20, Ebola was confirmed by reverse transcription–polymerase chain reaction (RT-PCR). Genomic sequencing of Ebola virus from her blood specimen identified six mutations not found in 25 other genomes sequenced from Liberia (5) or in 107 genomes obtained from Guinea, Mali, and Sierra Leone (6–8). The investigation found no history of travel by patient A, no interaction with visitors from Sierra Leone or Guinea, no recent funeral attendance, and no contact with a person with symptoms consistent with Ebola.

Patient A did report unprotected vaginal intercourse on March 7, 2015, with an Ebola survivor (survivor A), a man aged 46 years from another community in Monrovia. Survivor A had experienced onset of symptoms consistent with Ebola, including fever, anorexia, and headache on September 9, 2014, and was admitted to an Ebola treatment unit on September 23. His first test by RT-PCR on September 28, 2014, was indeterminate (positive on one assay with a cycle threshold of 40 indicating a low viral load and negative on a second assay). A second specimen was negative by RT-PCR on October 3, 2014. Survivor A was discharged from the Ebola treatment unit on October 7, 2014 and reported no subsequent illness or symptoms.

Survivor A had multiple family members with whom he lived or interacted with confirmed or suspected Ebola during the same period as his symptoms and Ebola treatment unit admission (Table). His older brother was confirmed with Ebola on September 5, 2014, from a postmortem blood specimen. Survivor A’s younger brother and daughter were admitted to an Ebola treatment unit on September 23, 2014, with symptoms consistent with Ebola. His younger brother died on September 25 and his daughter died sometime before September 28. No laboratory results were available for survivor A’s younger brother or daughter. Survivor A’s son entered a holding center on October 8, 2014, was confirmed to have Ebola on October 11 and died soon thereafter.

A new blood specimen was collected from survivor A on March 23, 2015, as part of patient A’s case investigation. The specimen was negative for Ebola virus by RT-PCR. Enzyme-linked immunosorbent assays for Ebola virus glycoprotein- and nucleoprotein-specific immunoglobulin G (IgG) antibodies were positive; immunoglobulin M (IgM) was undetectable. A semen specimen, collected from survivor A on March 27, 2015, was positive by RT-PCR with a cycle threshold of 32. Complete genome sequencing of the viral RNA from survivor A’s semen has not been possible to date given the low level of detectable viral nucleic acid. However, the partial sequence obtained so far (28% of the genome) closely matches the sequence from patient A. A rapid diagnostic test was conducted to evaluate human immunodeficiency virus (HIV) as a possible reason for long-term viral shedding. The HIV test was negative.

In addition to patient A, survivor A reported recent unprotected vaginal intercourse with a woman aged 45 years (contact A) with no history of illness. Intercourse with contact A occurred on three to five occasions between the last week of February and March 15, 2015. A blood specimen collected from contact A on March 27, 2015 was negative for Ebola virus–specific IgG and IgM.

Since January 21, 2015, all new confirmed cases of Ebola in Liberia have been epidemiologically linked to a single transmission chain (CDC Liberia Ebola Response Team, unpublished data, 2015). Ebola viral RNA from three of the 22 confirmed cases in this transmission chain (with onset dates of January 8, January 27, and February 9, 2015) were sequenced and compared with the genetic material from patient A. None of the sequences from these isolates shared the mutations observed in patient A’s isolate.

## Discussion

Available epidemiologic and laboratory findings indicate that patient A may have been exposed to Ebola virus through sexual contact with survivor A, whose semen was PCR-positive 199 days (September 9, 2014 to March 27, 2015) after his likely Ebola onset. Although the diagnostic RT-PCR in September was indeterminate, survivor A’s positive enzyme-linked immunosorbent assays, specifically against the viral nucleoprotein, indicate previous Ebola virus infection. His clinical course and epidemiologic links suggest that he had Ebola in early September 2014. The diagnostic tests were performed 18 and 24 days after symptom onset, and the results may have reflected convalescence. Although less likely, it is also possible that his Ebola virus infection occurred later and the indeterminate test result reflected the absence of Ebola virus in September 2014.

Ebola virus RNA in survivor A’s semen in March 2015 does not prove the presence of infectious virus. However, the absence of patient A’s genetic signature in sequenced RNA from three patients in Liberia’s last known cluster of epidemiologically-linked cases makes it unlikely that patient A was infected from unrecognized, ongoing community transmission. Culture of survivor A’s semen specimen for Ebola virus is planned to determine whether viable virus was present.

It is not possible to definitively ascribe Ebola infection in patient A to transmission from survivor A, and another sexual partner or other source cannot be excluded. However, the timing of intercourse between survivor A and patient A, the subsequent illness in patient A, the presence of viral RNA in survivor A’s semen, matching genetic sequences (where coverage has been obtained) in isolates from survivor A and patient A, and the lack of other known exposures suggest possible sexual transmission. Enrichment methods are being applied to survivor A’s semen sample to amplify existing Ebola virus RNA and complete genomic sequencing. Other limitations of the investigation include 1) the relatively small number of sequenced genomes from Ebola patients in this epidemic, which limits an assessment of the generalizability of the molecular findings; and 2) incomplete laboratory results and Ebola treatment unit and hospital records for some of survivor A’s family members, preventing confirmation of Ebola and exact dates of death.

Previously, CDC and WHO recommended abstinence or condom use for at least 3 months following recovery from Ebola. However, to prevent transmission of Ebola, contact with semen from male survivors should be avoided. If male survivors have sex (oral, vaginal, or anal), a condom should be used correctly and consistently every time until further information is known. Used condoms should be handled and disposed of safely to avoid contact with semen. After handling of condoms, or following any physical contact with semen, skin should be washed thoroughly with soap and water. Based on information from this investigation, CDC, the World Health Organization, and the Government of Liberia issued updated recommendations for survivors (4,9,10).

Investigations of several other recent Ebola cases in West Africa have suggested sexual transmission from survivors but have not been confirmed (CDC Emergency Operations Center, unpublished data, 2015). Additional studies are planned to determine clearance, persistence, and shedding of Ebola virus in body fluids of survivors and to evaluate possible sexual transmission of infection. Use of RT-PCR testing of semen (e.g., evidence of two negative tests) might be a useful tool for assessing and counseling male survivors on measures they should take to prevent transmission of Ebola virus. CDC and other public health partners are reviewing existing data to determine the validity and feasibility of potential recommendations.

What is already known on this topic?

Ebola virus persists in seminal fluid following recovery, but the duration of viral shedding and the likelihood of sexual transmission are not known. Earlier studies have demonstrated that the virus can be isolated from semen as long as 82 days after symptom onset, and that semen can be positive by reverse transcription–polymerase chain reaction, indicating presence of viral RNA, up to 101 days after onset. Possible sexual transmission was reported in 1968 for Marburg virus, a related filovirus, but has not been clearly documented for Ebola.

What is added by this report?

Ebola virus can persist in the seminal fluid of convalescent men for longer than previously recognized and can potentially lead to sexual transmission of Ebola.

What are the implications for public health practice?

Until more information is known, contact with semen from a male survivor should be avoided. If male survivors have sex (oral, vaginal, or anal), a condom should be used correctly and consistently every time. Additional studies are planned to examine Ebola virus persistence in body fluids of male and female convalescent patients and the likelihood of sexual transmission.

Transmission of Ebola in West Africa has diminished over the past few months. However, awareness of possible sexual transmission from survivors to partners and the importance of prevention measures is needed. Sufficient supplies of condoms and counseling to promote their correct and consistent use should be provided as part of the response in Ebola-affected countries. In addition, efforts should be undertaken to prevent the possibility of sexual transmission from stigmatizing survivors.

## Figures and Tables

**TABLE:** Course of Ebola in survivor A and family members — Liberia, 2014

Relationship to survivor A	Age (yrs)	Date of symptom onset	RT-PCR results	Test dates	Date of death
Brother	62	August 22	Positive	September 5	Unknown (before September 5)
Brother	36	September 9	Not done	—	September 25
Survivor A	46	September 9	Indeterminate	September 28	Living
			Negative	October 3	
Daughter	14	September 16	Not done	—	September 23–28
Son	12	October 2	Positive	October 11	Unknown

**Abbreviation:** RT-PCR = reverse transcription–polymerase chain reaction.
